# Comparison of rectal volume definition techniques and their influence on rectal toxicity in patients with prostate cancer treated with 3D conformal radiotherapy: a dose-volume analysis

**DOI:** 10.1186/1748-717X-4-14

**Published:** 2009-05-11

**Authors:** Cem Onal, Erkan Topkan, Esma Efe, Melek Yavuz, Serhat Sonmez, Aydin Yavuz

**Affiliations:** 1 Department of Radiation Oncology, Baskent University Medical Faculty, Adana, Turkey

## Abstract

**Background:**

To evaluate the impact of four different rectum contouring techniques and rectal toxicities in patients with treated with 3D conformal radiotherapy (3DCRT).

**Methods:**

Clinical and dosimetric data were evaluated for 94 patients who received a total dose 3DCRT of 70 Gy, and rectal doses were compared in four different rectal contouring techniques: the prostate-containing CT sections (method 1); 1 cm above and below the planning target volume (PTV) (method 2); 110 mm starting from the anal verge (method 3); and from the anal verge to the sigmoid flexure (method 4). The percentage of rectal volume receiving RT doses (30–70 Gy) and minimum, mean rectal doses were assessed.

**Results:**

Median age was 69 years. Percentage of rectal volume receiving high doses (≥ 70 Gy) were higher with the techniques that contoured smaller rectal volumes. In methods 2 and 3, the percentage of rectal volume receiving ≥ 70 Gy was significantly higher in patients with than without rectal bleeding (method 2: 30.8% vs. 22.5%, respectively (p = 0.03); method 3: 26.9% vs. 18.1%, respectively (p = 0.006)). Mean rectal dose was significant predictor of rectal bleeding only in method 3 (48.8 Gy in patients with bleeding vs. 44.4 Gy in patients without bleeding; p = 0.02).

**Conclusion:**

Different techniques of rectal contouring significantly influence the calculation of radiation doses to the rectum and the prediction of rectal toxicity. Rectal volume receiving higher doses (≥ 70 Gy) and mean rectal doses may significantly predict rectal bleeding for techniques contouring larger rectal volumes, as was in method 3.

## Background

Prostate cancer is a radio-responsive tumor with a well-defined dose-response relationship [1,2]. Higher radiotherapy (RT) doses have been associated with better biochemical control rates and fewer distant relapses [1,3]. Those findings support the suggestion that enhanced survival rates may be achievable with an improvement in local control. However, the use of higher RT doses is limited by an increased risk of complications in adjacent normal tissues. In this setting, more sophisticated techniques such as three dimensional conformal RT (3DCRT), intensity modulated RT (IMRT), and tomotherapy allow more precise treatment planning with better sparing of the normal tissues [4], which yields higher local control with significant reduction in both acute and late complications[5] Nevertheless, the use of higher RT doses beyond the conventional doses has been demonstrated to cause a moderate increase in the dose-limiting, late rectal toxicity, mainly manifested by rectal bleeding [6,7].

The major predictor of rectal bleeding is the volume of the rectum included in the high dose region [8,9], and the correlation between rectal bleeding rates and the irradiated rectal volume has been well established [9-12]. Furthermore dose-volume histograms (DVH) served as useful tools in demonstrating this significant relationship. Despite its extreme importance, no universally accepted method has been established for rectal contouring in RT planning for prostatic carcinomas. The length of rectum contoured has been defined in different ways by different authors. Examples of these definitions include: 1 cm above and below the planning target volume (PTV)[13,14], the length of the rectum in prostate-containing tomography sections [15], 110 mm of rectum starting from anal verge [12,16], or the anal verge to the rectosigmoid flexure [10,17-19].

One important drawback of using different rectal definitions and contouring methods is the resultant difficulty in interpreting the outcomes of different studies. Thus, we planned to compare four different rectal volume definition techniques and dependent irradiated percent rectal volumes on predicting rectal toxicity in patients with localized prostate cancer treated with 3DCRT, which will be a guide for evaluating the rectal toxicity wherein the rectal contouring technique used.

## Methods

### Patient Data

A total of 118patients with histological proof of prostate adenocarcinoma was treated with 3D-CRT between January 2007 and February 2008 in the Department of Radiation Oncology at Baskent University. We analyzed clinical and dosimetric data of 94 eligible patients. Eligibility criteria were as follows: Eastern Cooperative Oncology Group performance status (PS) of 0 to 2; age between 18 and 70 years; non-prostatectomised; no prior chemotherapy or abdominal irradiation; no distant metastasis; no contraindication for RT. Invariably, all eligible patients were treated with the same technique and the same doses, and any deviations from either the technique or dose were reasons for exclusion from the study. The clinical and dosimetric records of patients with stage T1c-T3 (American Joint Committee on Cancer, 1997 staging system) prostate cancer were used in this analysis. Prostate specimens were scored with the Gleason grading system. According to our current protocol, all patients were treated with 3 months of neoadjuvant total androgen blockage prior to planned irradiation. Baskent University's Institutional Review Board approved this study design.

### Treatment Planning

As part of treatment planning, all patients underwent a CT scan with 2.5-mm slice thickness. During the scan, patients were in supine with their feet fixed in a commercially available knee support device, an emptied rectum, and comfortably full bladder. Patients were asked to empty their rectum before treatment, no enema or other laxatives were used before planning CT and during treatment. The CTV was defined as the entire prostate and seminal vesicles. A 1-cm margin was added to the CTV to define the planning target volume (PTV). The treatment volume included an additional 0.7-cm margin for beam penumbra in all directions, except for the posterior margin, which overlaps the rectum; thus, posteriorly, a 0.5-cm margin was added for reducing rectal toxicity. The isocenter was positioned in the center of the PTV and beams were shaped with multi-leaf collimators (MLC; Varian DHX 3323, Varian Medical Systems, Palo Alto, California, USA).

The exposed rectum was defined in four different ways for all 94 patients as depicted in Table 1. All target and organ at risk volumes were defined and contoured by the same physician. Intra-observer variability was also assessed on randomly selected 10 sample patients by a blind repetition of rectum contouring on randomly chosen CT scans. The mean intra-observer variability was 0.7 mm in the cranial and 0.9 mm in the caudal directions, respectively.

**Table 1:** Rectum contouring techniques

Methods	Techniques
1	All prostate-containing CT sections
2	1 cm above and below PTV-containing sections
3	110 mm of rectum starting from the anal verge
4	Anal verge to the sigmoid flexure

All treatments were planned with a six-field technique using a treatment planning system (Eclipse^®^, Varian Medical Systems, Palo Alto, California, USA). A total of 70 Gy (2 Gy/fr, daily, Monday through Friday) was delivered using 18-MV photons. Portal images obtained from the anterior set-up and two lateral fields on the first treatment day and once weekly, or more (if necessary), during the RT period, were used to confirm field verifications by comparing them with digitally reconstructed radiographs. The portal images were reviewed by the treating physician.

### DVH Analysis

The dose distribution of each plan for each patient's rectum was established and the doses were re-calculated for the different rectal volumes lengths. The DVHs created for each patient and methods were used to perform inter-method comparisons. Our analysis included the percent volume of rectum irradiated with certain dose levels (30 to 70 Gy, in 10 Gy increments) evident on DVHs created for each method, and their possible predictive role on rectal toxicity incidence and severity. All doses represent total doses that have not been corrected for fractionation.

### Toxicity Score

Side effects manifested within 90 days from the initiation of RT were considered "acute", and "late" those manifest thereafter. Rectal toxicities were graded according to the Radiation Therapy Oncology Group (RTOG) toxicity scores [20]. The rectal toxicity grades are: grade 1 = minor symptoms requiring no treatment; grade 2 = symptoms that respond to simple management; grade 3 = distressing symptoms affecting lifestyle and necessitating hospital admission; grade 4 = symptoms necessitating a major surgical procedure (laparatomy, colostomy, long stay in hospital); and grade 5 = death. Grades 1 and 2 rectal bleeding is defined as incidental or intermittent bleeding requiring no treatment or responding to simple outpatient management, respectively; grade 3 rectal bleeding is defined as bleeding that requires a blood transfusion or laser cauterization.

During the RT course, all eligible patients were evaluated on the same day of the week for toxicity scoring, unless a patient required more frequent visits. In the medical records, the type of toxicity and its grade, the time of occurrence, as well as the prescribed medications and doses were systematically reported.

### Follow-up

The length of follow-up was calculated from the first date of 3DCRT. According to the medical records, follow-up visits included a thorough physical examination, serum total and free prostate specific antigen (PSA), and testosterone levels, complete blood count and serum biochemistry, and pelvic MRI every 6 months. At each visit, detailed genitourinary and gastrointestinal system toxicities were assessed. The patients were first seen 6 weeks after the completion of RT and every 3 months or more frequently, if necessary, thereafter.

### Statistical Analysis

The dosimetric variables considered were rectal volume, maximum and mean dose to the rectal volume (Dmax and Dmean, respectively), and volumes (percentage and absolute) of rectum receiving 30 Gy, 40 Gy, 50 Gy, 60 Gy, and 70 Gy. For each patient and each technique, DVHs were compared for both dosimetric assessment and their predictive value on rectal toxicity. The Fisher's exact test was used to compare qualitative variables and the Student's *t *means comparison test was used for continuous variables. The median values of these differences were compared using the Wilcoxon signed rank test to evaluate if they were significantly different from zero. A *p *≤ 0.05 (two-sided) was considered significant for all statistical tests.

## Results

Total 94 of 118 eligible patients were evaluated. 26 patients were excluded from the study, because, 16 patients were treated after radical prostatectomy, 6 patients were treated with pelvic box technique because of lymph node metastasis, and 2 patients did not finish the sheduled treatment (1 with myocardial infarction, 1 with no reason). The patient and disease characteristics are summarized in Table 2. All 94 patients were eligible for toxicity analysis and no patient was lost to follow-up; the median follow-up interval was 13.1 months (range: 3–21.6 months). The treatment protocol was well tolerated in general with no report of grade 4 or 5 acute or late toxicity. Sixteen patients (17%) completed the treatment without any significant complications. Rectal toxicities of grade 1 to 3 were reported in 34 (36%), 36 (38%), and 8 (9%) patients, respectively. Rectal bleeding was reported in 13 (14%) patients, and were graded as grade 2 in 12 (13%), and grade 3 in the remaining 1 (1%). This latter patient was presented at the 9^th ^month after 3DCRT and fared well following two courses of laser cauterization.

**Table 2:** Patient characteristics.

Patients	
Age (years)	
Median	69
Range	48–82
Pretreatment PSA	**n (%)**
≤ 10 ng/mL	37 (39)
**> **10 ng/mL	57 (61)
Gleason score	**n (%)**
6	55 (59)
7	30 (32)
8	3 (3)
9	6 (6)
Stage	**n (%)**
T1	12 (13)
T2	63 (67)
T3	19 (20)

The median prostate and seminal vesicle volumes were 38 cm^3 ^(range: 18–111.7 cm^3^) and 13 cm^3 ^(range: 4.8–28.8 cm^3^), respectively. The median prostate, seminal vesicle, and PTV doses were 69.7 Gy (range: 68.5 – 71.3 Gy), 69.8 Gy (range: 68.4 – 72.0 Gy), and 70.0 Gy (range: 68.7 – 71.5 Gy), respectively.

Table 3 shows the median rectum volumes using the different contouring techniques. As expected, compared to methods 1 and 2, relatively larger rectum volumes were contoured in methods 3 and 4. Table 4 shows the comparison of rectum minimum, maximum and mean doses and percentage of rectal volumes receiving different doses based on these different techniques. The mean rectal doses and percentage of rectal volumes receiving different dose levels were higher with contouring techniques that resulted in small rectal volumes (method 1) than with contouring techniques that resulted in average and large rectal volumes (method 3). Thus, for example, the mean rectum dose and V70 were higher in method 1 (57.5 Gy and 32.9%, respectively) than in method 3 (49.6 Gy and 24.3%, respectively).

**Table 3:** The Median Rectum Volumes

Methods	Volume in cm^3 ^(min-max)
	Rectum
1	43.6 (22.0–147.3)
2	54.7 (29.8–161.4)
3	63.0 (36.5–175.3)
4	60.5 (30.5–176.2)

**Table 4:** Median Dose-Volume Histogram and Dose-Wall Histogram data for the patients treated with 3DCRT.

	Method 1	Method 2	Method 3	Method 4	*p*
Min dose (Gy)	8.2	3.2	1.5	1.9	0.01
Mean dose (Gy)	57.5	54.0	49.6	51.1	<0.001
Max dose (Gy)	75.1	75.1	75.1	75.1	NS
V30 Gy (%)	86.7	84.5	80.6	82.1	<0.001
V40 Gy (%)	78.1	73.3	68.6	70.7	<0.001
V50 Gy (%)	70.9	64.2	55.3	59.8	<0.001
V60 Gy (%)	55.6	48.4	41.7	45.1	<0.001
V70 Gy (%)	32.9	27.9	24.3	26.5	<0.001

The comparison of rectal minimum and mean doses, V30, V40, V50, V60 and V70 Gy revealed significantly higher doses for method 1 compared to the other methods; the lowest mean rectal doses and percentage of rectal volumes at different dose levels were obtained with the technique used in method 3. The minimum and mean rectal doses significantly differed in each method. Similarly, statistically significant differences were established for the percentage of rectum volumes receiving 30 Gy, 40 Gy, 50 Gy, 60 Gy, and 70 Gy, respectively.

Acute rectal toxicity was closely associated with the mean rectum doses and V30 Gy, V40 Gy, V50 Gy, V60 Gy and V70 Gy points for all contouring techniques. As shown in Table 5 mean rectal doses and V70 Gy were significantly higher in patients with Grade 2 or more rectal toxicity compared to patients with or without Grade 1 rectal toxicity. The mean rectal dose in patients with Grade 2 or more rectal toxicity was lowest in method 3 (52.4 Gy) and highest in method 1(61.0 Gy). Likewise V70 Gy values were higher in methods 1 and 2 (42.3% and 37.3%) compared to methods 3 and 4 (32.5% and 33.4%), respectively.

**Table 5:** Mean rectal doses and percentage of rectal volume receiving 70 Gy (V70 Gy) values according to acute rectal toxicity grade groups.

	Mean Rectal Dose (Gy)			**V70 Gy (%)**		
	Grade 0–1	Grade ≥ 2	*p*	Grade 0–1	Grade ≥ 2	*p*
Method 1	53,4	61,0	< 0.001	27,2	42,3	< 0.001
Method 2	49,8	58,4	< 0.001	23,0	37,3	< 0.001
Method 3	44,5	52,4	< 0.001	19,8	32,5	< 0.001
Method 4	46,1	54,7	< 0.001	20,7	33,4	< 0.001

When rectal bleeding was evaluated Wilcoxon test revealed that, in method 2, the percentage of rectal volumes those received ≥ 70 Gy were 30.8% and 22.5% for patients with and without rectal bleeding (*p *= 0.03), respectively. Similarly in method 3, the percentage of rectal volume that received ≥ 70 Gy was 26.9% and 18.1% in patients with and without bleeding (*p *= 0.006). The mean rectal dose was found to be a significant predictor of rectal bleeding only in method 3; mean rectal doses were 48.8 Gy and 44.4 Gy for patients with and without bleeding (*p *= 0.02). No significant correlation was found for low or moderate dose levels.

## Discussion

In this study, four different rectum contouring techniques were assessed, and the impact of the contouring techniques on DVH and acute rectal toxicity and rectal bleeding was evaluated. We clearly demonstrated that mean rectal dose and rectal volume receiving a high dose (≥ 70 Gy) are the most important predictive factors for acute rectal toxicity and rectal bleeding, and varies according to rectal contpuring techniques. This significance was assessed in this study with different rectum contouring techniques, and especially the method 3 revealed a significant correlation.

The primary aim of 3D-CRT in prostate carcinoma is to maximize the therapeutic ratio to deliver an effective dose to the tumor while maintaining an acceptable dose to the neighboring normal tissues. In this manner, better control of the local tumor and reduction of distant metastatic rates can be achieved by escalating the dose beyond that of conventional doses without additional toxicities [9,21,22]. However, toxicities such as late rectal bleeding, which is one of the dose-limiting complications, may prevent escalation of the dose and therefore adversely affect treatment outcomes. The volume of the rectum included in the high dose region is the major determinant for predicting late rectal bleeding. In recent years a number of studies evaluated the relationship between rectal toxicity and rectal irradiation, and for this purpose rectal DVHs, dose wall histograms (DWHs), and dose surface histograms (DSHs) have been used. However, the definitions of the affected rectum varied widely among the researchers [10,12-14,16-19,23], and no universally accepted, conclusive result has been obtained with respect to whether DVH, DSH, or DWH is the best predictor of rectal complications, including late rectal bleeding. Nor has such a result been obtained to determine which length of the contoured rectum provides the best predictor of complications. In this current study, we compared mean rectal doses and percentage of rectal volumes receiving particular doses (30–70 Gy) via DVHs in most commonly used four rectal contouring techniques to an effort to determine the best contouring technique for prediction of rectal toxicity.

The use of different rectal contouring techniques with different rectal lengths and volumes yield various radiation doses, which may result in a variety of toxicity probabilities. This issue has been addressed by various authors. One of the most important predictors of acute rectal toxicity and rectal bleeding is the rectal volume receiving a high dose (60–80 Gy) [19,24,25]. Koper et al. found that the risk of rectal bleeding increased from 10% to 63% when the irradiated rectal volume increased from 25% to 100% [17]. In that study, the rectum was contoured from the anal verge proximally to the sacroiliac joint. Michalski et al., in the preliminary report of toxicity from an intergroup trial, observed that the relative risk of developing late gastrointestinal system toxicity was two-fold greater if the total rectal volume receiving radiation dose exceeded 100 cm^3^; the rectum was contoured as a solid organ extending from the anus to the rectosigmoid flexure [21]. In a randomized trial, Pollack et al. reported a significant increase in rectal toxicity in patients treated with 78 Gy compared to 70 Gy [16]. The DVH calculations were performed with respect to the rectal volumes within a 11-cm cranio-caudal segment, with no specification as to whether the rectal contents were included. The authors demonstrated that the 5-year risk of grade ≥ 2 rectal toxicity was 37% in patients with > 25% of the rectum receiving ≥ 70 Gy compared to 13% for patients with < 25% of the rectum receiving ≥ 70 Gy. In addition, all grade 3 complications occurred when V70 exceeded 30% of the rectal volume [9]. In this current study, we clearly demonstrated that all grade ≥ 2 acute rectal toxicities were seen in patients with > 30% of the rectum receiving ≥ 70 Gy regardless of contouring techniques (Table 5). Also a significant correlation was found between rectal bleeding and rectal volume receiving ≥ 70 Gy for rectum contoured in methods 2 and 3.

The mean rectal dose is another dosimetric factor that predicts rectal morbidity. Zapatero et al. demonstrated that the mean rectal dose and V60 Gy were closely correlated with grade 2 or worse rectal bleeding in 107 patients with prostate cancer treated with 3DCRT [25]. They found that patients with rectal bleeding had a mean rectal dose of 57 Gy compared with 46 Gy for those without bleeding (*p *< 0.0005). The rectum was contoured over 150 mm, from the anus (at the level of the ischial tuberosities) to where the rectosigmoid flexure could be identified. In the current study, we found a statistically significant correlation between rectal bleeding and mean rectal doses only when the rectum was contoured over 110 mm starting from the anus (method 3: 48.8 Gy for patients with rectal bleeding and 44.4 Gy for patients without rectal bleeding (*p *= 0.02)). The fact that this correlation was significant only in method 3 may be due to the fact that this technique contours larger rectal volumes than the other techniques that we used. The rectum contoured in the study of Zapatero et al. was even longer and the rectal volume larger compared to those in method 3 of our study. Thus, mean rectal doses may significantly predict rectal bleeding for techniques contouring larger rectal volumes.

In one of the first studies that evaluated the rectal contouring problem, Geinitz et al. concluded that a uniform definition of the rectal volume should be established to achieve equivalent DVH results [26]. Boehmer et al. compared two different rectal contouring techniques: one technique included the rectum bounded by two CT slices above and below the PTV; the other technique included the rectum from the anal verge to the sigmoid colon [27]. Furthermore, the posterior half of the rectum was contoured for both volumes. The first technique resulted in significantly higher minimum and mean rectal doses than did the second technique. The authors concluded that different ways of rectal contouring significantly influence calculated doses to the rectum. In another study, Liu et al. compared 6 different ways of contouring the rectum in 10 patients with prostate cancer treated with a four-field box-technique with a total dose of 70 Gy. They concluded that absolute rectal wall volume, in addition to percent rectal volume, should be used in analyzing late rectal toxicity [24].

Our study also demonstrates that the rectal DVHs vary considerably with different rectum delineation techniques. The rectum contoured in all prostate-containing CT sections (method 1) had the largest percentage of rectum receiving a specific radiation dose, since less rectum volume was contoured. Any contouring techniques that use a longer length of the rectum will result in a smaller percentage of the contoured rectum receiving the radiation dose. Thus, the technique that contoured a 110-mm rectal segment from the anal verge (method 3) resulted in lower radiation doses than the techniques that contoured shorter segments and smaller rectal volumes. This is due to the fact that the absolute volume of rectum receiving a specific dose remains constant while the percentage of rectal volume receiving a specific dose becomes reduced if the total volume contoured is larger. Therefore, with different rectal length and volume contouring techniques, the differences in the configurations of the different DVHs become apparently significant.

## Conclusion

In conclusion, with a relatively larger patient population, we demonstrated that percentage of rectal volumes receiving high doses (≥ 70 Gy) and mean rectal doses which are predictors of rectal toxicity varied in different rectum contouring techniques with differing DVHs. The rectal volume exposed to high RT doses (≥ 70 Gy) seems to be a crucial determinant in predicting late rectal bleeding in almost all contouring techniques. In method 3; in which rectum was contoured 110 mm starting from anal verge, rectum volume was found to be higher than other methods, and a significant importance of mean rectal dose and percentage of rectal volume receiving >70 Gy was established. Finally, we think that, there is an urgent need for a universally accepted precise definition of rectal volumes for a systematic reliable comparison of various histograms.

## Competing interests

We have no personal or financial conflict of interest and have not entered into any agreement that could interfere with our access to the data on the research, or upon our ability to analyze the data independently, to prepare manuscripts, and to publish them.

## Authors' contributions

All authors read and approved the final manuscript. CO prepared the design of the manuscript and made the contouring of the target volume and organs at risk; ET and MY collected the samples; AY gave advise on the work and helped in the interpretation of the data; EE and SS made the treatment planning; CO wrote the paper together with ET.
